# Comparative molecular developmental aspects of the mammalian- and the avian lungs, and the insectan tracheal system by branching morphogenesis: recent advances and future directions

**DOI:** 10.1186/1742-9994-9-16

**Published:** 2012-08-07

**Authors:** John N Maina

**Affiliations:** 1 Department of Zoology, University of Johannesburg, Auckland Park 2006, P.O. Box 524, Johannesburg, South Africa

**Keywords:** Lung, Development, Tracheal system, Branching morphogenesis, Growth factors

## Abstract

Gas exchangers fundamentally form by branching morphogenesis (BM), a mechanistically profoundly complex process which derives from coherent expression and regulation of multiple genes that direct cell-to-cell interactions, differentiation, and movements by signaling of various molecular morphogenetic cues at specific times and particular places in the developing organ. Coordinated expression of growth-instructing factors determines sizes and sites where bifurcation occurs, by how much a part elongates before it divides, and the angle at which branching occurs. BM is essentially induced by dualities of factors where through feedback- or feed forward loops agonists/antagonists are activated or repressed. The intricate transactions between the development orchestrating molecular factors determine the ultimate phenotype. From the primeval time when the transformation of unicellular organisms to multicellular ones occurred by systematic accretion of cells, BM has been perpetually conserved. Canonical signalling, transcriptional pathways, and other instructive molecular factors are commonly employed within and across species, tissues, and stages of development. While much still remain to be elucidated and some of what has been reported corroborated and reconciled with rest of existing data, notable progress has in recent times been made in understanding the mechanism of BM. By identifying and characterizing the morphogenetic drivers, and markers and their regulatory dynamics, the elemental underpinnings of BM have been more precisely explained. Broadening these insights will allow more effective diagnostic and therapeutic interventions of developmental abnormalities and pathologies in pre- and postnatal lungs. Conservation of the molecular factors which are involved in the development of the lung (and other branched organs) is a classic example of nature’s astuteness in economically utilizing finite resources. Once purposefully formed, well-tested and tried ways and means are adopted, preserved, and widely used to engineer the most optimal phenotypes. The material and time costs of developing utterly new instruments and routines with every drastic biological change (e.g. adaptation and speciation) are circumvented. This should assure the best possible structures and therefore functions, ensuring survival and evolutionary success.

## Introduction

"*‘Specifically, convergence provides a way to tell features that have important functional significance from features that do not.’ Vogel*[1]*.*"

The development of the vertebrate lung was a decisive preparatory event for adaptation to air-breathing and successful transition from water to land, momentous occasions in the evolution and diversification of animal life (e.g. [2-4]). Cost-effective acquisition of molecular oxygen (O_2_) allowed accomplishment of more energetic lifestyles like flight (e.g. [5-7]) which lead to marked adaptive radiation and speciation (e.g. [8,9]). Motley of conserved genes and molecular factors orchestrated proper transformations from simple to complex body forms by programmed organization and arrangement of cells and tissue components (e.g. [10-18]). Physiological processes (e.g. metabolic-, respiratory-, and ionic exchange) occur across two-dimensional (2D) surfaces which are increased by the three-dimensional (3D) assemblage of organs, tissues, and cells. Branched structures are common in nature and biology in particular. In the later, they include the conductive nervous tissue (e.g. axons and their arborisation), vascular system, vertebrate lungs, secretory glands, tubules of the kidney, and the insectan tracheal system (e.g. [19-24]). Mainly formed to secrete and/or transport materials/fluids or conduct/transfer ions and effects like electric activity, e.g., actions potentials in nervous tissue, branched systems comprise of distinctively hierarchically arranged structural parts (segments = domains) which interconnect in a coherent manner. The overall morphology granted by the frequency and the geometry of bifurcation. In tubular branched systems, depending on the type of organ, endothelial- (in the vascular system) or epithelial cells (in all the other organs) line the internal space (cavity/lumen). The similarities amongst the various branched biological structures underscore existence of shared programmed regulatory mechanisms which involve signaling molecules, transcription factors, and other growth instructing molecular agents which instruct the growth and bifurcation patterns (e.g. [15,16,21,23]). Identification and characterization of the morphogenetic drivers which prompt budding and bifurcation from pre-existent domains is pivotal to determining the control and the regulation of the process of branching morphogenesis (BM). The controlling mechanism of BM involves dualities of morphogenetic factors: an agonist is regulated by an inhibitor and *vice versa* (e.g. [16,21,25-29]). There is mounting evidence that combinatorial action of various signaling molecules, transcription factor families, and other molecular factors is vital to cell specification, differentiation, and tissue development. For example, interactions between fibroblast growth factor (FGF) signaling and Wnt/*β-*catenin signaling in the lung mesenchyme positively reinforce each other (e.g. [17]), BMP-4 counteracts the effects of FGF-10 [30]; De Langhe et al. [31,32], mesenchymal FGF signaling is needed for the expression of Wnt-2a and mesenchymal Wnt/*β-*catenin signalling [33,34], and mesenchymal Wnt/*β-*catenin signaling is required to sustain expression of FGFR-1 and FGFR-2 and GATA-6 and Nkx2.1 operate in a synergistic way to instruct pulmonary epithelial differentiation and development [35]. Well-coordinated spatiotemporal expressions and repressions of the morphogenetic factors initiate, hone, and optimize the ultimate phenotypes (e.g. [36-38]). For the murine lung, comprehensive gene expression profiling using oligonucleotide-based microarrays showed that ∼ 11,000 genes are expressed throughout the developmental stages of the lung [36].

**Figure 1:**
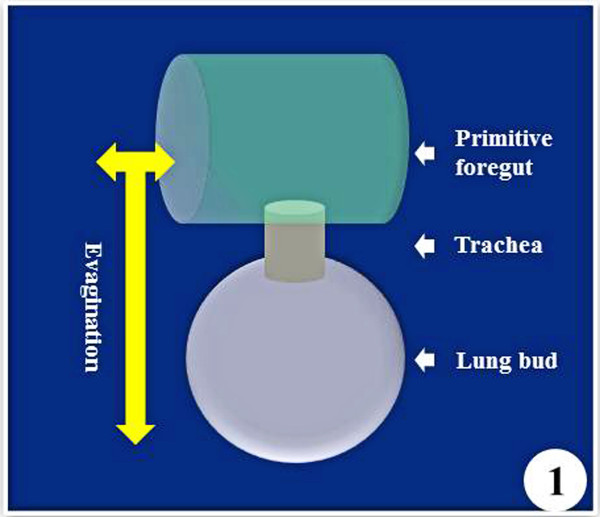
**The lung bud and subsequently the lung inaugurates by evagination (out-pocketing = diverticulation) of committed endodermal cells of the primitive foregut.** Its growth and development is regulated by various molecular morphogenetic factors that are expressed at different times and places in the epithelial, mesenchymal, and mesothelial compartments.

The embryonic development of the vertebrate lung inaugurates with a ventral out-pocketing (evagination) of dedicated (committed) cells from the formative primitive foregut endoderm into the splanchnic mesenchyme (Figure 1) (e.g. [39-42]) and interactions between cells originating from two germ layers - endoderm and mesoderm (e.g. [42-45]). The lung buds elongate and branch to form the trachea and the main bronchi followed by stereotypic branching and budding which produces the conducting airways, leading to the alveolar region of the peripheral lung. Among others, these changes prepare the lung for air-breathing in the postnatal life. The distinctive structural features of lungs are large surface area, intense vascularization (which generates large capillary blood volume), and thin blood-gas barrier (e.g. [46,47]). Large respiratory surface area emanates from intense internal subdivision of the lung, a phenotype fashioned by different morphogenetic cues that are expressed in form of growth factors (GFs) and cytokines, molecular instruments which act as paracrine signals that control cell division and differentiation (e.g. [21,39,48]).

In evolutionary terms, many developmental pathways are conserved in the animal kingdom (e.g. [13,49-55]): the common origin of these processes is strongly implied in the biological past. In the developing lung bud, epithelial cells of the airways and endothelial ones of the vasculature multiply rapidly while the formative structures undergo reiterative branching, producing a highly ordered arrangement (e.g. [45,56]). While a lot still remains to be resolved, the signals and the manner in which they are regulated during BM are becoming clearer (e.g. [16,21,23,40,57]). Detailed insights into the mechanisms of BM will not only increase our understanding of the development of the lung but will further advance techniques of lung engineering and regenerative medicine (e.g. [58,59]), design of artificial organs to replace failed/failing ones (e.g. [60]), and therapeutic interventions, especially during early stages of lung development (e.g. [61-65]). More importantly, insight into the process of BM of one kind of branched organ should permit meaningful understanding of others. For an area that is receiving intense scientific interest and scientific accounts (publications) are appearing prodigiously, occasional critical reviews of the subject matter are necessary to identify what is certain, what is speculative and therefore ambiguous, and where gaps in knowledge exist. Heuristic collation and reconciliation of available information should help highlight areas for further research, helping avert improvident and costly duplication of effort by investigators. Presently, much of the investigative activity of the genetic- and molecular aspects of the development of gas exchangers in particular and branched structures in general is on the mammalian lung (particularly on those of laboratory animals - mainly the mouse and rat) and the tracheal system of insects (especially of *Drosophila*). Relatively little information exists on the morphogenetic aspects of the development of the avian lung and hardly any is available on the amphibian- and reptilian ones. Holistic understanding of the mechanisms involved in the process of BM of the gas exchangers will only be accomplished once these gaps are closed. Moreover, although instructive in their own right, the shortcomings that are inherent in *in vitro* studies and those involving genetically manipulated (engineered) animals should be appreciated. A first in comparatively integrating the available data, this account succinctly outlines the process of BM and that of the development of the mammalian- (bronchioalveolar) and avian (parabronchial) lungs as well as that of the insectan tracheal system, the only taxa where meaningful data are presently available.

## Branching Morphogenesis (BM)

Branched structures are ubiquitous in nature. They occur at every scale and form of development in both the plant- (e.g. [66,67]) and the animal kingdoms (e.g. [52,67]). The design of branched forms has constantly fascinated biologists, mathematicians, and physicists (e.g. [67-72]). A prototypical developmental process, BM is mechanistically fabricated by few simple iterative genetic subroutines through which complex well-ordered, functionally efficient architecture is engineered [73]. An assemblage described as ‘growth and branching of epithelial buds’ by Saxena and Sariola [74] and ‘creation of branched structures’ by Davies [67], in animal tissues and organs, BM occurs in the lung (e.g. [20,21,25,53,75-79]), glandular organs like the mammary gland, the salivary gland, and the pancreas (e.g. [28,80-83]), the kidney (e.g. [22,84-86]), the tooth [87], the tracheal system of insects (e.g. [23,88,89]), and the vasculature (e.g. [19]). In most cases, the functional units (e.g. secretory or gas exchange units) display distinctive 3D architecture (e.g. [30,79]). Organs that form by BM provide good models for studying and understanding application of the mode of development in animal patterning, cell differentiation, and organ and tissue organization (e.g. [15,45,90,91]). Branched structures form by coordinated spatiotemporal expression of specified morphogenetic cues [25,92].

**Figure 2:**
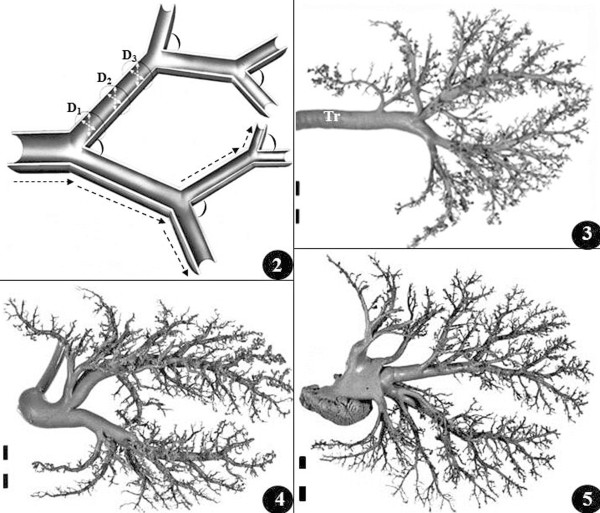
**A generic diagram of branched tubular system.** By specifying where signaling molecules, transcription factors, and other molecular factors are localized or expressed, the lengths, the diameters, and the sites where branches form and the angles of bifurcation are specified. Dashed arrows show the lengths of the constituent segments; D_1_-D_3_, axial diameters (small dashed arrows) at various points of the length of a part; the arcs show the angles of bifurcation. Figures 3,4,5: Latex cast preparations of the airway (Figure 3), the venous (Figure 4), and the arterial- (Figure 5) systems of a mature lung of the pig (*Sus scrofa*). Product of complex branching process, the systems match to optimize gas exchange between air and blood. Tr, trachea. Scale bars: 1 cm. From Maina and van Gils [115].

**Figure 3:**
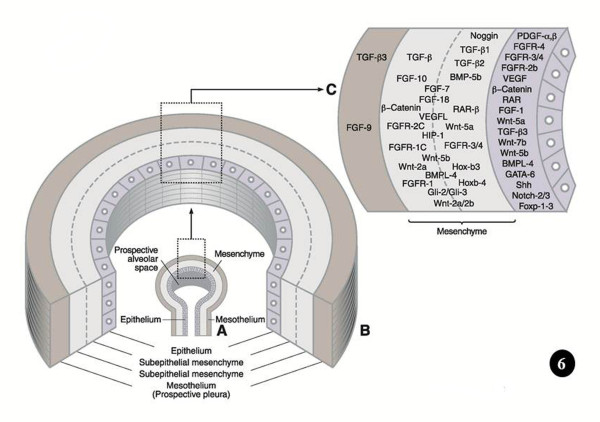
**Figure**6**A-C. Sites where key signaling molecular factors, transcription factors, and other molecular agents are expressed and/or localized to regulate the development and growth of the lung.** In the mesenchyme, the areas of expression are not exact.

**Figure 4:**
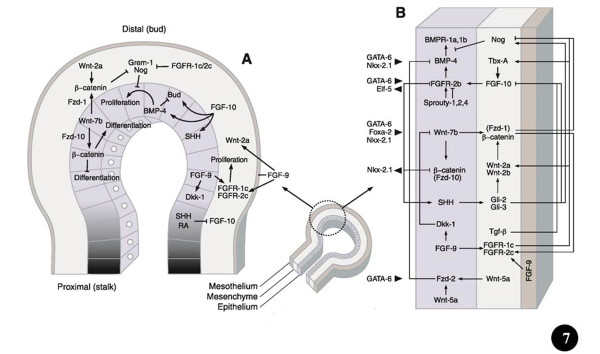
**Ways and means by which prominent signaling molecules and transcription factors control lung development.** The middle (small) diagram shows the different parts (germ layers) of a lung bud (proximal and distal). Figure 7A: Location of signalling molecules and the pathways by which they control growth, budding, and differentiation of the airway epithelium and surrounding mesenchyme. The shading shows the proximal-distal extent of the lung bud epithelium. Figure 7B: Signaling programs in the distal lung bud bring about mesenchymal- and epithelial cell proliferation as well as airway branching. Details to these processes can be obtained from the text and particularly from the following comprehensive reviews: Metzger and Krasnow [25], Perl and Whitsett [11], Roth-Kleiner and Post [116], Cardoso and Lü [40], Lu and Werb [53], De Langhe and Reynolds [117], Affolter et al. [77], Warburton et al. [21], [15,16,18,29,98]. Modified after Ornitz and Yin [18].

**Figure 5:**
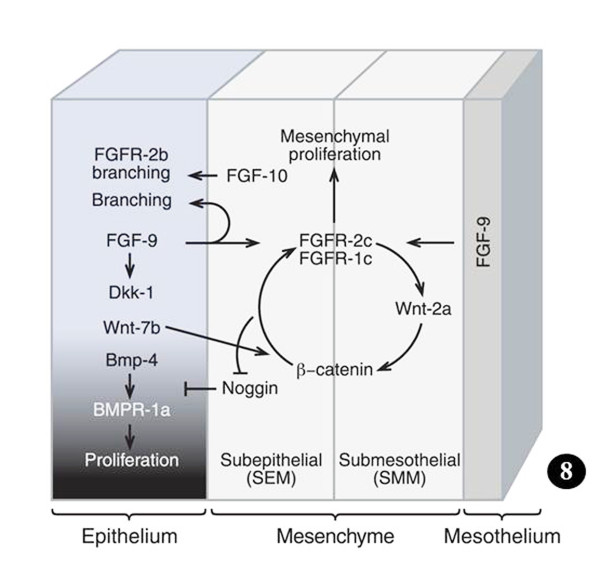
**Figure 8: Feed-forward signaling in which FGF-9 controls Wnt-2a expression and mesenchymal Wnt/β-catenin signaling, and in which mesenchymal Wnt/β-catenin signaling is requisite for mesenchymal FGFR expression and mesenchymal responsiveness to FGF-9.** In absence of either FGF-9 or canonical Wnt ligands, the mesenchymal FGF-9-Wnt/β-catenin feed-forward network breaks up, occasioning loss of mesenchymal FGFR expression and FGF responsiveness. Wnt/β-catenin signaling has to be preserved in order to induce or maintain FGFR expression, FGF-9 responsiveness, and continuance of FGF-Wnt/β-catenin feed-forward signaling. Loss of mesenchymal FGF-Wnt/β-catenin signaling increases Noggin expression in both the subepithelial and submesothelial mesenchyme. Lack of either pathway decreases mesenchymal proliferation. The change in the density of the shading of the epithelium shows the proximal-distal extent of the lung bud. Details can be acquired from Yin et al. [33], Rajagopal et al. [118], Yi et al. [119] De Langhe et al. [32], and Ornitz and Yin [18]. Modified after Ornitz and Yin [18].

Normal lung development culminates in formation of airways and blood vessels which branch (Figure 2), pattern, and closely relate to each other (Figures 3,4,5): this increases the respiratory surface area and reduces the diffusion distance for molecular oxygen (O_2_) between inhaled air and capillary blood. Also, proper geometries and sizes of the airways and the blood vessels grant optimal (cost-effective) flows of the respiratory fluid media, saving on energy required to transport them through the conduits (e.g. [93]). While the iterating process involved in BM may appear deceptively simple to genetically program, the instructions and the molecular factors that drive it are profoundly intricate (e.g. [20,21,23,94,95]). BM is driven by an assortment of genes and intercellular signaling molecules that include transcriptional factors, soluble peptide growth factors, and insoluble extracellular matrix molecules that are expressed in the right quantities, time, place, and sequence. This determines the points where new branches form, the lengthening of the intervening duct/trunk/stalk before downstream branching occurs, and where groups of cells detach from the epithelium of the main duct to form side branches (secondary budding) (e.g. [16,18,21,25,39,40,79,96-98]). In computer lexicon, a highly specific genomic information flow engine initiates and regulates spatiotemporal expression and transcription of appropriate morphogenetic cues which produce a protocol-based pulmonary architecture by programming and reprogramming branching periodicity and bifurcation angles. Additionally, physicochemical and environmental cues and factors like intraluminal hydraulic pressure (e.g. [21,99,100]), relative hypoxia (e.g. [101]), and calcium concentration (e.g. [102,103]) play important roles in BM. ‘Cross-talk’, i.e., cell-to-cell signaling, especially between the mesenchymal- and the epithelial cells (e.g. [85,91]), is necessary to correctly educe cell-specific developmental pathways that lead to proper lung development and differentiation of various epithelial cell lineages (e.g. [104-106]). In the adult human lung, e.g., after 20–23 bifurcations, a highly ordered system of airways with ~25,000 bronchioles (e.g. [45,107]) gives rise to ~300 to ~600 million alveoli (e.g. [108-110]): a respiratory surface area of ∼ 140 m^2^ exists [111]. More than 40 different types of cells (pneumocytes) exist in the lung (e.g. [109,112-114]).

**Figure 6:**
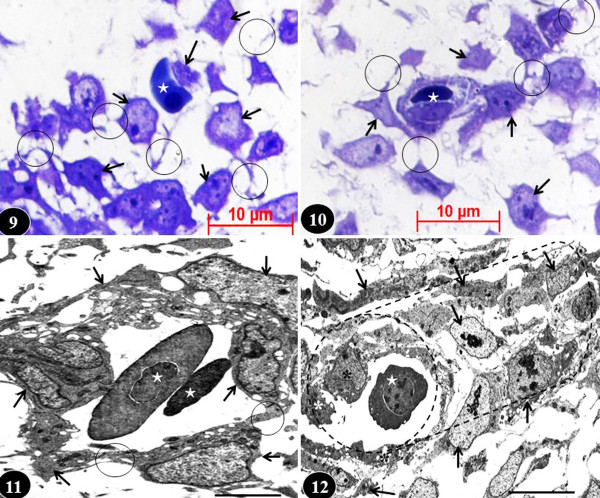
**Developing chicken lung.** Figure 9: Radical transformation of mesenchymal cells into characteristically stellate angioblasts (arrows) with conspicuous filopodia (circles) (which allow them to move) and into hemopoietic cells, i.e., red blood cells (star), at the fourth day of embryonic lung development. Toluidine stained section. Figure 10: Angioblasts (arrows) moving to surround a red blood cell (star) on the fifth day of embryonic lung’s development to form a blood vessel. Circles, filopodia. Toluidine stained section. Figure 11: Angioblasts (arrows) surrounding red blood cells (stars) in the developing lung on the sixth day of embryonic life forming a blood vessel. Circles, filopodia connecting angioblasts to form the vessel lumen. Figure 12: A blood vessel with a well-defined lumen in the lung of a seven day old embryo. Arrows, angioblasts forming the wall; asterisks, endothelial cells; star, red blood cell; dashed cylindrical shape, orientation of the blood vessel. These changes occur under the influence of VEGF. Figures 11 and 12 are transmission electron micrographs. Scale bars: Figure 11, 10 μm; Figure 12, 15 μm.

**Figure 7:**
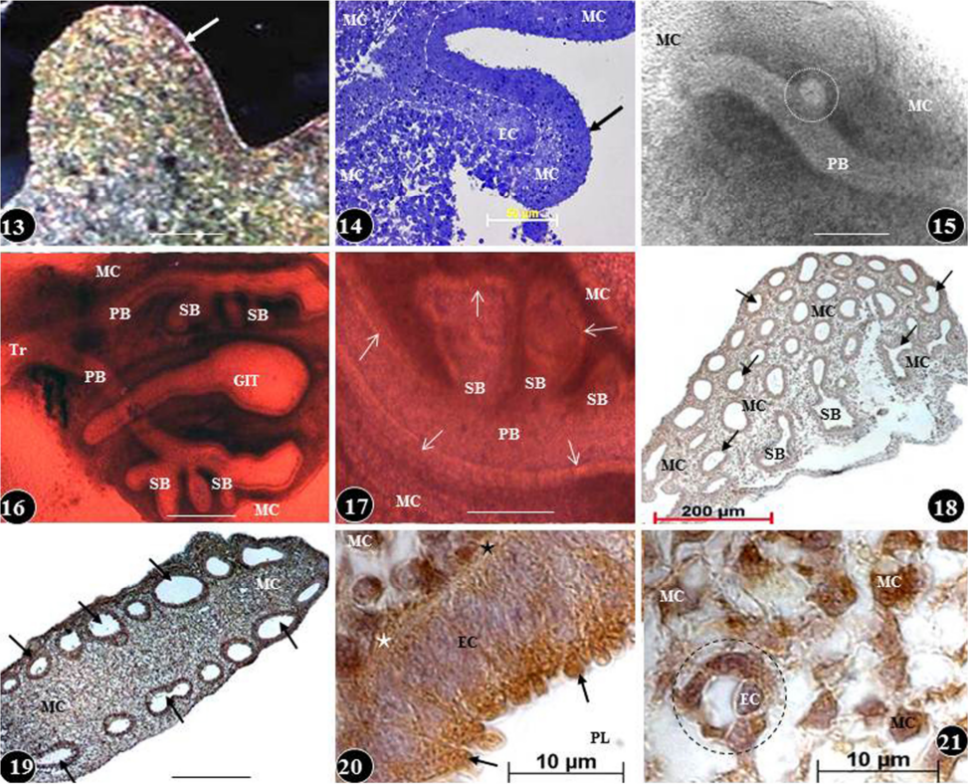
**Early development of the chicken lung (Figures 13–17) and expression of basic FGF (bFGF-2) at different stages of development (Figures 18–21).** Figures 13 and 14: Lung buds (arrows) on the third- (Figure 13) and fourth days (Figure 14) of embryonic life: initially, the bud comprises of a rather homogenous group of cells (Figure 13) while a little later the epithelial cells (EC) and the mesenchymal cells (MC) reorganize into separate areas (dashed lines). Toluidine blue stained preparations. Scale bars: Figure 13, 100 μm; Figure 14, 50 μm. Figures 15–17: Matrigel cultured lung buds. On the fourth day of embryonic life (Figure 15), a secondary bronchus (dashed circle) can be seen sprouting from a primary bronchus (PB) and at the fifth day of life (Figure 16), extrapulmonary primary bronchi (PB) can be seen continuing into the developing lung and giving rise to secondary bronchi (SB). Tr, trachea; GIT, developing part of the gastrointestinal system. MC, mesenchymal cells. Figure 17: Close-up of developing airways at the sixth day of embryonic life. The epithelium lining the primary bronchus (PB) and the secondary bronchi (SB) can be seen (arrows). Figures 16 and 17 are colour adapted images of black and white microscopic originals. Scale bars: Figure 15, 200 μm; Figure 16, 200 μm; Figure 17, 0.2 mm. Figures 18 and 19: Transverse- (Figure 18) and longitudinal (Figure 19) sections of developing chicken lungs at the sixth day of development showing expression of basic fibroblast growth factor-2 (bFGF-2) in the mesenchymal cells (MC) and in the epithelial cells lining the airways (arrows). SB, secondary bronchi. Scale bar: Figure 19, 100 μm. Figures 20 and 21: Expression of bFGF-2 in the parabronchial epithelial cells (EC) (Figure 20) and in the mesenchymal cells (MC) (Figure 21) of the lung of a seven day old embryo. bFGF is overexpressed in the apical parts of the epithelial cells (arrows) (Figure 20) and in the mesenchymal cells (Figures 20 and 21). PL, parabronchial lumen; stars, basement membrane of epithelial cells; circle, developing blood vessel; EC, endothelial cell. Figure 13 is from Maina [132]; Figure 13 is from Maina [133]; other figures-unpublished.

**Figure 8:**
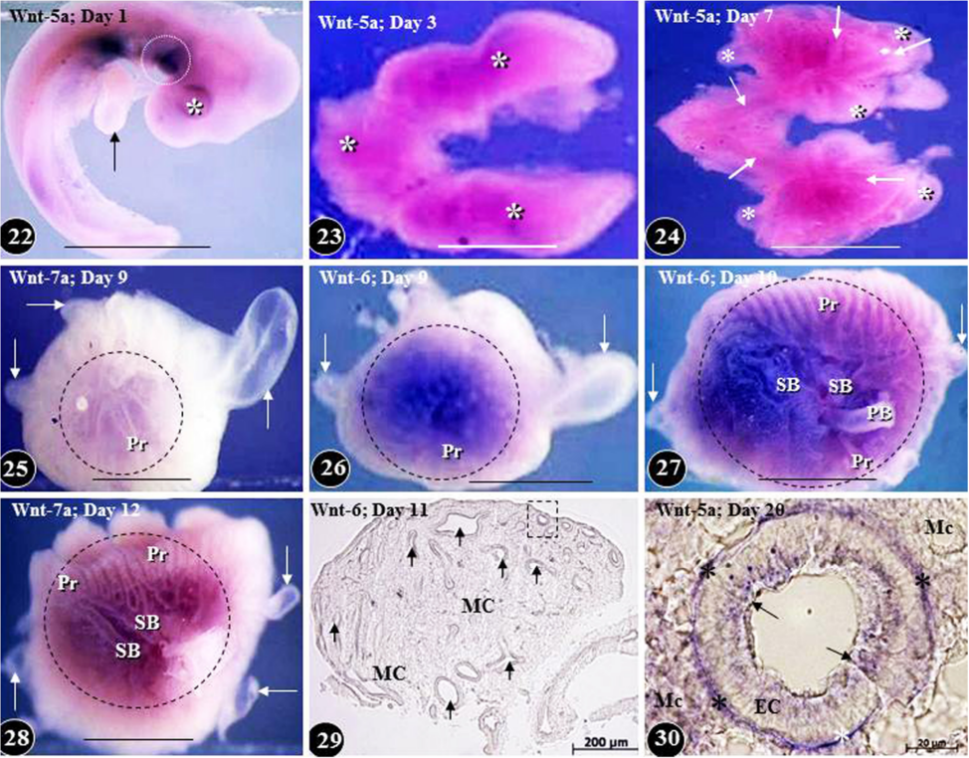
**Expression of Wnt signaling molecules in developing chicken embryo (Figure 22) and in the lung at different times of embryonic life (Figures 23–30). Wnt-5a is expressed on the ventral aspect of the body trunk at day two of life (Figures 22).** Arrow, developing fore limb bud; asterisk, optic placode; dashed circle, prospective site of the development of the lung and the heart. Scale bar, 1 mm. Figures 23: Diffuse expression of Wnt-5a (asterisks) in the developing lung on day three of life. Scale bar, 1 mm. Figure 24: Expression of Wnt-5a in developing lung on day five of life. Wnt-5a is predominantly localized in the developing airways (arrows) and relatively little of it in the mesenchyme and the air sacs (asterisks). Scale bar, 1 mm. Figures 25–28: Expression of Wnts in developing lungs at different embryonic days of life (areas marked by dashed circles). Figures 25, 26, and 28 show lateral aspects of the lung while Figure 27 is that of a medial one. The Wnts are poorly expressed in the developing air sacs (arrows). PB, primary bronchus; Pr, parabronchi; SB, secondary bronchi. Scale bars, 1 mm. Figure 29: Transverse section of a developing lung at the eleventh day of life showing Wnt-6 expression in the epithelium lining the airways (arrows) and the mesenchyme (MC). An airway like the one enclosed in the dashed square is shown enlarged in Figure 30. Figure 30: Cross-section of a parabronchus showing the expression of Wnt-5a at the 20^th^ day of development. Wnt-5a is highly expressed in the epithelial cells (arrows), the mesenchymal cells (MC), and in the basement membrane of the epithelial cells (stars). Figures 22–28 are from Maina [132]; Figure 30 is from Maina [134]; Figure 29 is from unpublished work of RG Macharia and JN Maina.

Evolution causes selection pressure to conserve functionally important coding and regulatory pathways (e.g. [120]). The genes that are involved in BM are conserved across animal species (e.g. [23,25,52,67,79]). In computational jargon, these elements are ‘hard-wired’. The genetic instructions that lead to analogous designs have been continued over long evolutionary time scales. By among others Wagner [121,122], Wallace [123], and Mojica et al. [124], such constant and ubiquitous forms, structures, and systems have been termed ‘Bauplans’ (= ‘builder’s plans’ = ‘blue-prints’ = ‘frozen an excellent example of such conserved construct. The transformation of the unicellular organisms (protozoa) to multicellular organisms (Metazoa), which occurred in the Ediacaran era, ~500 to 700 million years ago (mya), is the original branching morphogenetic process (e.g. [125-128]). The genes involved in BM can be traced back to a common origin - a set dedicated to regulating pattern formation [129]. Much of the genomic reconfiguration occurred during the Cambrian Explosion, ~530 million years ago, when massive increase in animal body plans occurred and phenomenal speciation happened (e.g. [9,130]). The genes that encode for BM appear to have developed gradually: Pavlova et al. [75], e.g., showed them to correlate with the expression of different, but interrelating, genomic subgroups, signifying differences in morphogenetic mechanisms at the various stages in evolution of branching tubules. In signaling biology, which entails transduction and transcriptional controls, few canonical developmental programs are exploited more frequently across species, tissues, and stages of elaboration (e.g. [52]). Based on the notion that important regulatory pathways are commonly genetically conserved among species, comparative genomics approach has been used to identify well-conserved controlling factors (e.g. [120,131]). For the development of the lung, among others, the best known genes, molecular factors, and regulatory pathways are the Bone Morphogenetic Proteins (BMPs), the Fibroblast Growth Factors (FGFs), Sonic Hedgehog (Shh), Wnt genes/proteins (Wnts), Transformation Growth Factors (TGFs), Retinoic Acid (RA), Vascular Endothelial Growth Factor (VEGF), and Extracellular Matrix (ECM) component proteins. Most of these instruments have been shown to be involved in the formation of the insectan tracheal system. The morphogenetic factors are succinctly outlined below. The sites of localization and expression of signaling molecules, transcription factors, and other morphogenetic molecular cues, which regulate the development of the lung bud, are shown in Figure 6 and the mechanisms by which they achieve it are outlined in Figures 7 and 8. Table 1 summarizes the areas of expression of some of the lung’s signaling molecules, transcription factors, and other molecular factors and the phenotypes specified by genetic mutations, targeted inhibition, abrogation, blockage or under expression of certain genes and their products (proteins).

**Table 1:** Expression patterns of some lung signaling molecules, transcription factors, and other molecular factors and the phenotypes specified by their mutations, targeted inhibition, abrogation, blockage or underexpression

**Signaling molecule**				
	**Gene name**	**Expression site**	**Phenotype**	**References**
FGF-18	Fibroblast growth factor-18	Mesenchyme	Poor alveolization	Usui et al. [135]
FGF-9	Fibroblast growth factor-9	Epithelium; mesothelium (future pleura)	Little airway branching; mesenchymal hypoplasia	Colvin et al. [136]; Yin et al. [17]
PDGF-a	Platelet derived growth factor-a	Epithelium	Compromised myoblast and elastin formation; defective alveolization	Bostrom et al. [137]; Buch et al. [138]
Notch-2/3	Notch gene homologue-2/3	Epithelium	Defective alveolization	Xu et al. [139]
FGFR-3/4	Fibroblast growth factor receptor-3/4	Epithelium; mesenchyme	Deficient production of elastin; lacking alveolization	Weinstein et al. [140]
BMP-4	Bone morphogenetic protein-4	Epithelium; Mesenchyme	poor lung development with cystic terminal air spaces	Bellusci et al. [141]; Weaver et al. [142]; Jang et al. [143]
FGFR-2b	Fibroblast growth factor receptor-2b	Epithelium	Lung agenesis; flawed branching; poor epithelial cell differentiation	De Moerlooze et al. [144]; Shu et al. [145]
FGF-10	Fibroblast growth factor-10	Mesenchyme	Lung agenesis and different abnormalities	Sekine et al. [146]; De Moerlooze et al. [144]; [147]
WNT-5a	Wingless-related MMTV integration site-5A	Mesenchyme; epithelium	Excessive branching; tracheal deficiency	Li et al. [148]
WNT-7b	Wingless-related MMTV integration site-7B	Epithelium	Vascular scantiness; reduced mesenchymal proliferation	Shu et al. [149]; Shi et al. [150]; Mucenski et al. [151]
TGF-3β	Transforming growth factor-β3	Epithelium; mesothelium	Anomalous airway branching; poor extent of alveolization	Kaartinen et al. [152]; Shi et al. [153]
Shh	Sonic hedgehog	Epithelium	Poor bronchial branching; hypoplastic lungs; tracheoesophangeal fistula	Litingtung et al. [154]; Minoo et al. [155]
HIP-1	Hedgehog interacting protein-1	Mesenchyme	Defective branching	Chuang and McMahon [156]; Chuang et al. [26]
Catnnb-1	*β-*catenin	Epithelium	Impaired airway branching	Mucenski et al.[151])
VEGF	Vascular endothelial growth factor	Epithelium; mesenchyme	VEGF knockout have lethal phenotype; anormal vasculature	Zeng et al. [157]; Miquerol et al. [158]; Kasahara et al. [159]
Nog	Noggin	Mesenchyme	Lobation deficiencies	Weaver et al. [142]
**Transcription factors**				
FOXA-1/2	Forkhead box-A1/A2	Epithelium	Hypoplastic lungs; defective branching; poor smooth muscle formation	Wan et al. [160]
FOX-j1	Forkhead box-J1	Epithelium	Deficiency of ciliated cells; left-right asymmetry	Brody et al. [161]
FOX-F1a	Forkhead box-F1a	Mesenchyme	Defective branching and lobation; tracheoesophangeal fistula	Lim et al. [162]
HOX-A5	Homeobox-A5	Mesenchyme	Tracheal occlusion; poor airway branching	Aubin et al. [163]; Golpon et al. [164]
GATA-6	GATA-binding protein-6	Epithelium	Diminished airway branching; defective sacculation	Yang et al. [165]
Gli-2/3	GLI-Kruppel family member GLI-2/3	Mesenchyme	Lung agenesis	Motoyama et al. [166]; van Tuyl and Post [167]
Wnt-2/2b	Wingless-related MMTV integration site-2/2b	Mesenchyme	Lung agenesis	Harris-Johnson et al. [168]
NKX-2.1 (TTF-1)	Nkx homeodomain/thyroid-specific transcription factor	Epithelium	Lung agenesis	Stahlman et al. [169]; Kimura et al. [170]
HOX-a5	Homeobox-A5	Mesenchyme	Poor airway branching; thickening of the alveolar walls	Aubin et al. [163]
ALK-3	Aurora-like kinase	Epithelium	Impairment of lung branching; decreased cell proliferation	Sun et al. [171]
**Other molecular factors**				
Tmem 16a	Transmembrane protein-16a	Epithelium	Abnormal tracheal cartilage development	Rock et al. [172]
RARs	Retinoic receptors	Epithelium; mesenchyme; mesothelium	Lung agenesis and hypolasia; reduction in alveolar number; tracheoesophangeal fistula	Mendelsohn et al. [173]; Dickman et al. [174]; McGowan et al. [175]; Cardoso and Lü [40]
Itga-3	Integrinα-3	Epithelium	Defective branching	Kreidberg et al. [176]; De Arcangelis et al. [177]
Lama-5	Lamininα-5	Epithelium; mesothelium	Defective lobation	Schuger et al. [178]; Nguyen et al. [179]
Lmnb-1	Laminin-B1	Epithelium; mesenchyme	Impaired lobation	Schuger et al. [180]; Vergnes et al. [181]
PTHlh	Parathyroid hormone-like peptide	Epithelium	Impaired branching	Rubin et al. [182]

## Molecular aspects of the development of the Mammalian lung

### Bone Morphogenetic Proteins (BMPs) and Transforming Growth Factors (TGFs)

Out of the more than 20 family members, some BMPs have been shown to be directly involved in various developmental processes (e.g. [183]). Some like BMP-4, -5, and −7 occur in developing lung (e.g. [184]) where they control cell differentiation and proliferation in the epithelial lung buds. BMP-2 and −4 expressions are induced by FGF signaling [141,185]. Although the actual source of BMP-4 is controversial (e.g. [17,142]), it regulates epithelial proliferation through signaling to BMPR-1a (ALK-3) [171,185]. A recent study by Jang et al. [143], however, indicates that BMP-4 may only be expressed in the lung epithelium. It (BMP-4) has been shown to be involved in proliferation and survival of distal lung epithelial cells and in specifying development of smooth muscle cell precursors (e.g. [30,185-187]) and lung vasculogenesis and angiogenesis (e.g. [141,153,188,189]). BMP-5 is expressed across the embryonic lung mesenchyme and BMP-7 in the lung endoderm [190]. BMP-4 is similarly expressed by the proximate mesenchymal cells but its actual role there is unclear [77]: BMP-4 is an antagonist of FGF-10 [30]. Its expression decreases after the branching process has finished. This suggests that BMP-4 has negligible effect on the branching of the airways [141]. Null mutants of BMP-1 and −7 show no pulmonary defects. Unlike other BMPs, BMP-1 isn’t a member of the TGF-β Superfamily: it has a structure unique from the other BMPs and may be involved in activation of other BMPs [191,192].

Transforming growth factor-beta (TGF-β) family is a group of growth factors (GFs) (cytokines) which instruct lung development and play a pivotal role in instigation and pathogenesis of lung diseases [193-197]. The TGF-β family is part of a superfamily of proteins known as the TGF-β superfamily, which includes inhibins, activin, anti-müllerian hormone, BMP, decapentaplegic, and VG-1 [198,199]. Three isoforms of TGF-β, namely TGF-β1, TGF-β2, and TGF-β3, which utilize two receptors (TGFR-β1 and −2) have been reported in the mouse lung, with TGFR-β-2 being expressed only in the distal airway epithelium at early gestation (E11.5) and in both airway epithelium and mesenchyme from mid-gestation (E14.5) to postnatal day 14 [196]. Lack of TGF-β signaling causes abnormalities in BM and alveolization of the lung [194-196,200-202] while interestingly, excessive amounts of it causes serious hypoplasia in immature lung and fibrosis in the adult one [153]. This may occur from disruption of other peptide GFs which are involved in BM [203]. Chen et al. [196] noted that TGFR-β2 mediates TGF-β signaling and performs different roles in the lung epithelium and mesenchyme by differently controlling specific stages of lung development [196].

### Fibroblast Growth Factors (FGFs)

FGFs are multifunctional proteins with a broad variety of mitogenic, regulatory, morphological, and endocrine effects (e.g. [144,204]). Termed ‘pluripotent GFs’ and ‘promiscuous GFs’ (e.g. [205]) due to their multiple actions on many cell types, FGFs are involved in proliferation and differentiation of cells and tissues (e.g. [39,144,206]). They consist of a family of ∼ 23 gene encoding low molecular weight polypeptides with different developmental roles which include further cell growth and migration together with tissue repair, inflammation, angiogenesis, and tumour growth (e.g. [207-210]). They are heparin binding proteins and their interactions with cell-surface associated heparan sulfate proteoglycans have been shown to be required for FGF signal transduction. First isolated in pituitary extracts by Armelin [211], FGFs are the first angiogenetic factors to be sequenced [212]. Six members, namely FGF-1, -2, -7, -9, -10, and −18, are expressed in the lung (e.g. [140,213-216]). FGFs bind and signal via FGF tyrosine kinase receptors (FGFR1-5) which are expressed in the lung (e.g. [140,217,218]). They are mostly expressed in the pulmonary mesenchyme while their receptors are located in the lung epithelium. The exceptions are FGF-1 and -2 [(the acidic FGF (aFGF, FGF-1) and the basic FGF (bFGF, FGF-2, FGF-β)] which are expressed both in the fetal pulmonary epithelium and the mesenchyme [219,220]. FGF signaling, and specifically FGFR-3 and −4, are involved in the regulation of the basement membrane formation in the lung [36,221]. Interestingly, mutation of these two genes occasions failure of terminal lung development [36].

FGF-1 (aFGF) which instructs surfactant protein SP-B mRNA production stimulates epithelial cell proliferation which leads to formation, branching, and cell differentiation in the developing lung [204]. FGF-2 is a highly conserved GF which is generally involved in growth and development of different organs and tissues and induction of the mesoderm (e.g. [222-224]). It is a potent mitogen of the type-II pneumocytes [225] and has been associated with compensatory lung growth after damage from exposure to 95% O_2_[226]. FGF-2 is, however, an idiosyncratic GF: while it is produced in many cell types as well as in endothelial cells and cardiac myocytes and has been localized in cytoplasm, nucleus, and extracellular membrane (e.g. [227,228]), the actual mechanism by which it is secreted under normal physiological conditions is uncertain: it must have a consensus N-terminal signal sequence for its secretion [229].

Albeit the fact they possess some vascular and hematological defects, FGF-2 knockout mice are morphologically normal [230]. The disseminated expression of FGF-2 in the rat fetal lung, i.e., its localization both in the airway epithelial cells and the extracellular membrane (ECM) [219], resemble the pattern it presents in the avian lung [231]. One of the first genes which are upregulated in response to FGF-2 is Sprouty (Spry-2) [232,233]. Spry-2 negatively regulates FGF signal transduction by limiting or moderating the mitogen-activated protein kinase (MAPK) pathway (e.g. [232,234]): it (FGF-2) determines the site of production and hence the number of branches that develop in a certain domain [79]. FGF-1 and −7 produce different airway arrangements during pulmonary growth and development [204]. While FGF-7 is expressed very early in the mesenchymal cells of the developing lung (at sites where active branching occurs), its receptor (FGFR-2), is expressed only on epithelial cells [235]: it promotes mesenchymal-epithelial cell interactions [236]. According to Tichelaar et al. [237], during the development of the mammalian lung, FGF-7 is a more potent morphogen than FGF-10. In presence of other soluble factors, it (FGF-7) causes the trachea to transdifferentiate into distal lung, a transformation that FGF-10 doesn’t cause [90]: this shows that the developments of trachea and lung are regulated differently. *In vitro*, BM is disrupted by addition of exogenous FGF-7, antisense oligonucleotides, and neutralizing antibodies (e.g. [236,238]). In the mouse, the epithelial receptor for FGF-7 is the FGFR-2 IIIb isoform [144]: there is a lung phenotype when this isoform (but not another) is deleted. At day 10.5 of gestation, in the mouse lung, FGF-9 is abundantly expressed in the mesothelial cell layer and the epithelium [215]. It disperses to the mesenchyme to activate FGFR-1 signaling [239], ostensibly controlling the expression of mesenchymal genes, including FGF-10 [240]. FGF-9 performs functions of reciprocal epithelial-to-mesenchymal signaling and BM in the lung [17,215,241]. While many distal air spaces form and alveolar epithelial cell differentiation occur, FGF-9 null mice (FGF-9^−/−^) have severe lung hypoplasia and succumb in the prenatal stage [136,241]. Mouse embryos lacking FGF-9 show mesenchymal hypoplasia, diminished BM, and at the end of gestation, hypoplastic lungs that cannot support life [17]. Signaling concurrently, FGF-9 and Shh control growth and patterning of the pulmonary capillary plexes by regulating the expression of VEGF-A [242]. Together with *β-*catenin-mediated Wnt signalling, mesenchymal FGF-9 signaling acts in a feed-forward loop which sustains mesenchymal FGF receptivity and mesenchymal Wnt/*β-*catenin signaling [17]. FGF-9 largely signals to mesenchymal FGF receptors FGFR- 1 and −2 but also has the unique capacity of activating epithelial FGFR signaling [17,33,241,243].

Amongst all the FGFs expressed in the lung, only FGF-10 has been shown to be absolutely necessary to lung development (e.g. [184]). Through an intricate regulatory loop, it (FGF-10) regulates expression of BMP-4 at the growing (terminal) epithelial bud (e.g. [30,184,187]) while in response, BMP-4 [141], TGF-β1 [244], and Shh [245,246] (molecular factors expressed by the lung epithelial cells) limit FGF-10 production in the mesenchyme [39,91,247]. Up regulation of these factors in the very highly proliferative regions of the lung may stop or delay growth, induce quiescence, or promote lung bud maturation. Park et al. [235] attributed the formation of the airways largely to FGF-7 and FGF-10 and very little of it to FGF-2. During early lung development (branching period), FGF-10 is expressed in the mesenchyme at the distal tip of the new lung buds (e.g. [39,235,248,249]). From there, it spreads to activate FGFR-2b in adjacent epithelium, instructing a regular bifurcation pattern [116,187,246,250]. Appropriate spatiotemporal expression of FGF-10 is essential to correct organization of the lung epithelial tubules. Disruption of FGF-10-FGFR-2b signaling as well as overexpression of a dominant negative FGFR-2 in the mouse lung is lethal at birth. It causes multiple organ defects, including agenesis of the lung and termination of the trachea in a blind sac (e.g. [144,249,251]). FGF-10 plays a vital role in maintaining epithelial progenitor cell proliferation as well as co-ordination of alveolar smooth muscle cell formation and vascular development [147,187,251]. Furthermore, it (FGF-10) induces Shh, BMP-4, and Wnt-2 signaling, all of which are necessary for lung development (e.g. [30,146]). In the mouse, removal of FGF-18 gene has no specific effect on lung development [252,253]. However, FGF-18 knockout mice have decreased cell proliferation and alveolar spaces while overexpression causes asymmetric expansion of the conducting airways [135,254]. FGF-18 performs a vital role in lung alveolar development during late embryonic lung development but it is not directly involved in BM [216]. FGF signaling is mostly responsible for regulating mesenchymal proliferation while *β-*catenin signaling is an obligatory permissive factor for mesenchymal FGF signaling [17].

### Wnt growth factors/genes

The Wnt proteins, which are named in reference to the *Drosophila* gene *Wingless* and its mouse homolog *Integrase-1*, are a number of 19 family of secreted glycoproteins, signaling molecules which exert a broad range of important developmental processes (e.g. [42,98,255-257]). They produce morphogenetic effects by binding to cell surface receptor proteins (Frizzled), triggering a multi-step signaling cascade within the cell which allows *β-*catenin to move into the nucleus where it activates certain genes (e.g. [118,255,258-262]). By means of the canonical pathway, Wnt-2 and Wnt-2b signaling perform crucial and cooperative roles in determining lung endoderm progenitors within the anterior foregut, without affecting the specification of other foregut-derived tissues [42]: embryos lacking Wnt-2/2b expression present complete lung agenesis and don’t express Nkx2.1, the first marker of the lung endoderm. Also, Wnt proteins are profoundly involved in epithelial cell tubulogenesis in organs like lung, kidney, ear, mammary gland, gut, and heart (e.g. [261]). They regulate location and concentration of *β-*catenin, a protein which complexes with T-cell factor (TCF) in the nucleus: the complex (of *β-*catenin and TCF) activates the transcription of over 100 genes which perform various functions [263,264]. Wnt-*β-*catenin signaling is decisive to proper BM [118,265]: it refines the morphogenetic processes that are instructed by other upstream signaling pathways. Mesenchymal Wnt-*β-*catenin signaling controls FGFR-1 and FGFR-2 expression and consequently determines FGF signaling [33]. Wnt-5a and -7b are both expressed largely in the distal lung bud tip which is the site of most cell proliferation in embryonic lung [145,149,266,267]. Moreover, the signaling pathway regulates local specialization of the epithelium and the mesenchyme and the development of progenitor cell groups (e.g. [33,117]).

During the pseudoglandular stage of lung development, Wnt-2a and Wnt-7b are canonical Wnt ligands that actuate mesenchymal Wnt/*β-*catenin signaling while FGF-9 is the only ligand that signals to mesenchymal FGF receptors (FGFRs) [17]. Wnt-2 is expressed in the mesenchyme next to the tips of the airway buds [266,268]. This suggests presence of a relationship between Wnt expression and Shh signaling (e.g. [245]). During early lung development, Wnt-5a is expressed in both mesenchymal- and epithelial parts of the branching airways while in the pseudoglandular- and canalicular stages it localizes in the epithelium of the end-bud, with distinctive proximal-distal gradient [148]. Wnt-5a null mice evince increased cell proliferation both in the epithelium and the mesenchyme. This leads to growth of the distal lung, increased branching, and enlargement of the lung [148]. FGF-10, BMP-4, and Shh, which are all profoundly involved in BM, are expressed in Wnt-5a null mice [148]: Wnt-5a therefore acts as an inhibitory regulator of BM. Like the FGF-9 null mice, the only other mutant animal to display severe growth deficiencies [136], Wnt-7b null lungs are noticeably hypoplastic but show signs of normal patterning and cell differentiation [118]. Expressed only in the airway epithelium, with its highest levels occurring at the tips of the branching end-buds [266], Wnt-7b signals to adjacent cells to activate both autocrine and paracrine canonical Wnt signaling cascades. In Wnt-7b null mice, FGF-9 expression remains normal and both Wnt-7b and FGF-9 null mutants present reduced FGF-10 expression in the distal inter-bud region while normal expression in the proximal part of the lung bud occurs [118,241]. Concomitantly, these cascades provoke co-ordinated proliferation of contiguous epithelial- and mesenchymal cells to promote the growth of the organ, with limited changes in cell differentiation and morphogenetic patterning. Wnt-5a, a noncanonical Wnt, may disrupt the function of Wnt-7b by instructing and impeding lung growth [31,148]. Wnt-7b expression is regulated by TTF-1 (thyroid transcription factor-1), GATA-6, and FOXA-2, morphogenetic factors which are critical to proper lung development [169,266]. Mesothelial- and epithelial-derived FGF-9, mesenchymal Wnt-2a, and epithelial Wnt-7b have unique functions in the development of the mouse lung [17]: mesothelial FGF-9 and mesenchymal Wnt-2a are mostly in charge of supporting mesenchymal FGF-Wnt/*β-*catenin signaling while epithelial FGF-9 mainly affects epithelial branching.

### Sonic hedgehog (Shh)

Hedgehog (Hh) is a family of three secreted proteins termed Sonic hedgehog (Shh), Indian hedgehog (Ihh), and Desert hedgehog (Dhh) which play important roles in embryonic development. Amongst the Hh family, Shh, which is one of the morphogens involved in early lung development and is the best studied ligand (e.g. [269]): it (Shh) is expressed in the distal epithelium of the lung for the period of pseudoglandular stage of development. It produces its effects by binding to its receptors, patched-1 (Ptc-1) and Smoothen, transmembrane proteins that exist in contiguous sub-epithelial mesenchyme (e.g. [245,246,270]). Expressed at the tips of the end-buds, Shh negatively controls the distal mesenchyme FGF-10 expression, blocking lung bud extension while upregulating FGF-7 [246,247,271]. The zinc finger Gli genes are transducers of Shh signaling [272]. During the development of the lung, the genes are expressed in overlapping but well-defined areas of the mesenchyme [26,272-275]. Gli-2^(−1-)^ and Gli-3^(−1-)^ double mutant mice die by day 10.5 [166]: the lungs are hypoplastic, the right and left lobes don’t separate, and the tracheo-oesophangeal septum is defective, a phenotype which is similar to that displayed by Shh^(−/−)^[154] or TTF-1 (Nkx2.1)^(−/−)^ mice [155]. Mice with Gli-3 deficiency are viable but the lung is underdeveloped [273]. In Gli-2 null mutant mice, the tracheobronchial tube is not separated, the right and left lungs are connected, and the growth of the alveolar region is stunted [154,166,273]: the lung forms as one undersized lobe. Gli-1 double mutant mice have severe lung defects which are similar to those of the Shh^(−/−)^ mice, where the lung develops but BM is repressed [154]. Disruption of the membrane-bound Hedgehog interacting protein-1 (HIP-1) results in upregulation of Hh signaling, causing neonatal lethality from respiratory failure [156,276,277]: Hip-1 directly binds mammalian Hh proteins and moderates their signaling. Null mutation of Shh supresses lung epithelial branching [154,271]. In the mouse, conditional knockout of Shh in the lung epithelium generates fewer blood vessels and reduces VEGF expression [269]. Experimentally induced overexpression of Shh in the lung epithelium (using SP-C promoter) intensifies cell proliferation in both the mesenchyme and the epithelium while branching is not affected: it leads to development of superfluous mesenchyme and dearth of alveoli [246]. While FGF-10 doesn’t effect Shh expression, excessive amounts of FGF-7 suppress both Shh expression and signaling [235,247]. Shh- and FGF-9 signals control mesenchymal proliferation in specific submesothelial and subepithelial cellular compartments [241].

### Retinoic Acid

Vitamin A (retinol) brings about molecular signaling by the binding of its active metabolite (RA) to a group of heterodimerized TFs (transcription factors) [RA receptors α, β, and γ (RARα, -β, and -γ)] and retinoic-X receptors [α, β, and γ (RXR α, -β, and -γ)] (e.g. [278-280]). After RA binds, the nuclear receptors are activated and attach to their specific response sites in the promoter region of their target genes [281]. RA effects transcription of many genes and development and homeostasis in various organs, including the lung (e.g. [282]). It is expressed very early in lung development and continues throughout the process [283-285]. RAR-β is absent in the distal epithelium during BM but is expressed in the epithelial cells of the proximal- and the medium-sized airways while RAR-γ localizes mostly in the epithelium of the distal end-buds and demonstrates only weak expression in the proximal airway epithelium of the fetal- and adult lungs [282,286]. When RA is lacking during early stages of lung development (e.g. [282]), formation of oesophagotracheal septum is inhibited and the primary lung bud outgrowth doesn’t develop: it leads to lung agenesis or serious lung hypoplasia. Interestingly, upregulation of RA impedes BM while suppressing epithelial cell differentiation [282,286]. RA acts on cell programming and meaningfully instructs their differentiation [287]. Exogenous administration (*in vitro*) of RA upregulates FOXA-2 and TGFβ-3, two inhibitors of BM [287,288]. If RA signaling is blocked by a pan-RAR antagonist, expression of FGF-10, BMP-4, Shh, TTF-1, and GATA-6 is altered, prompting excessive airway branching [287,288]. Among the RA receptors, only signaling from RAR-β and RAR-γ is implicated in BM [282,286-288]. While RAR-β seems to impede branching, it is incontrovertibly involved in formation and stabilization of the conducting airways [282,286,288]. RA is vital in subdivision of the lung parenchyma [116,175,289]. Lungs of mice with obliterations of RAR-γ have less elastin and fewer alveoli [175] while RAR-α null mutant mice also have fewer alveoli [290]. Overexpression of dominant negative RAR-α in the mouse, just before and during alveolization, causes fewer but larger alveoli to form [291]. RAR-β signaling in the early postnatal period hinders alveolization [290,292]. Endogenous RA controls TGF-β activity in the prospective area where the lung forms, permitting local expression of FGF-10 and induction of lung buds Chen et al. [41].

### Extracellular matrix (ECM) component proteins

The ECM performs various roles in regulating cell function (e.g. [85]). It separates tissues, providing mechanical and structural support and/or offering a structure for cells to attach and or move on. Consisting of a collagen scaffold to which glycoproteins like tenasin, laminins, fibronectin, and proteoglycogens attach and intermingle with fibrinous proteins such as fibrillins and elastin, the ECM comprises of the basement membrane and the interstitial matrix (e.g. [293,294]). Signals transduced by α-3β-1 integrin may be involved in stimulating branch formation in the developing lung [176]. Matrix metalloproteinases perform important roles in remodeling the ECM (e.g. [295]). Absence or inhibition of interaction between epithelial cells with the basement membrane occasions failure of either normal lung development or lung injury repair [296]. Dearth of elastin decreases subdivision of the parenchyma in the mouse lung [297]. Elastin is important in alveolarization [298]. Suggestive of a prospective role in airway branching, tenascin-C accumulates in areas where new bronchial branches form [299,300]. Fibronectin expression increases to the highest level during airway branching [301,302]: it localizes in the mesenchyme at the epithelial-mesenchymal interface, commonly at points where airways bifurcate [302,303]. Inhibition of fibronectin matrix accumulation reduces BM [304]. The laminins are large multidomain glycoproteins that include three polypeptide subunits, namely α, β, and γ. Laminin α-1 is critical to lung BM and bronchial smooth muscle cell formation while laminin α-5 is necessary for normal lobulation and alveolization [180,305,306].

### Vascular Endothelial Growth Factor (VEGF)

**Figure 9:**
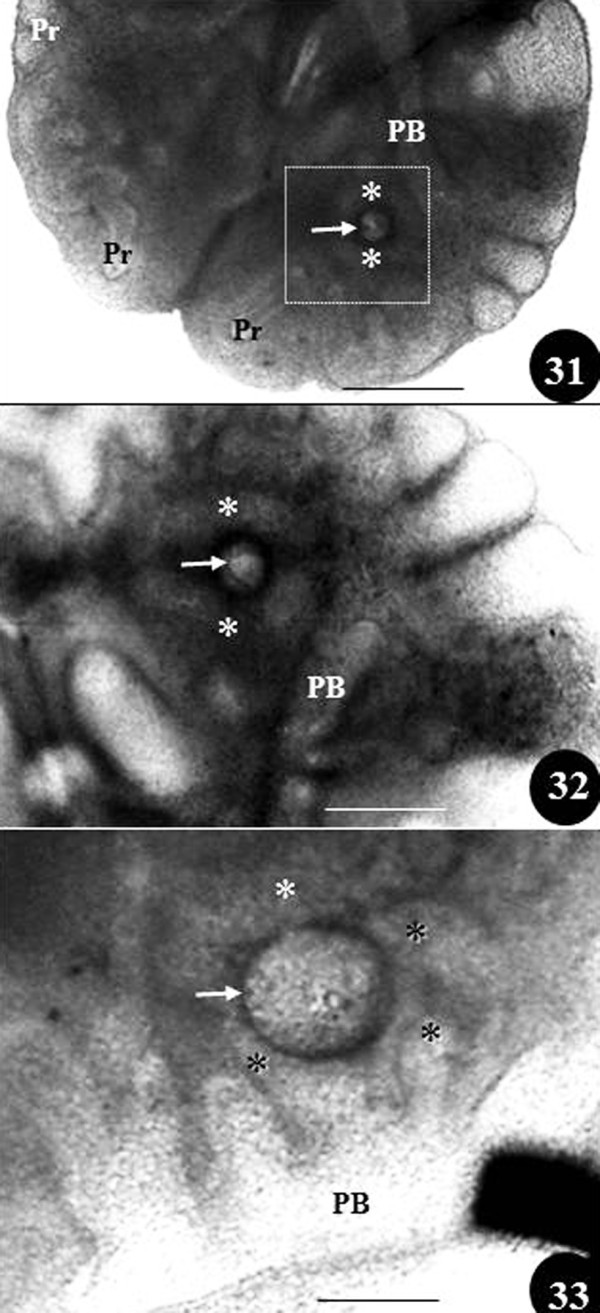
**Figure 31–33: Matrigel cultured preparations of developing chicken lungs showing effect of FGF-10 soaked in beads (arrows) on the developing secondary bronchi (asterisks) between days 6 (Figures 31, 32) and 10 (Figure 33) of embryonic life: Figure 32 is an enlarged view of the area enclosed in Figure 31.** The secondary bronchi (asterisks) are seen growing towards and surrounding the bead. PB, primary bronchus; Pr, parabronchi. Scale bars: Figure 31, 200 μm; Figure 32, 100 μm; Figure 33, 100 μm. From unpublished work done by JN Maina and B Kramer.

**Figure 10:**
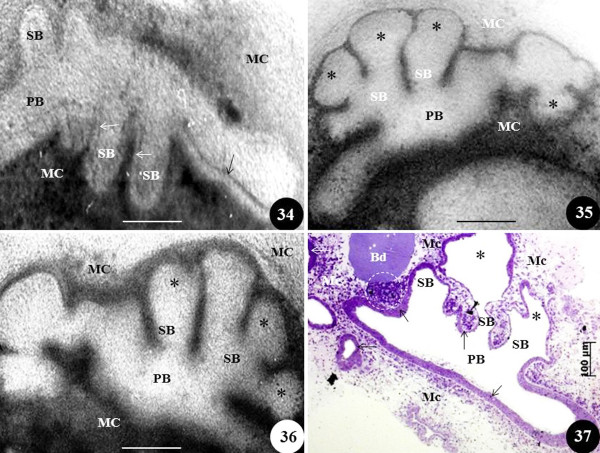
**Effect of exposure of excessive FGF-10 on matrigel cultured developing chicken lung.** Figure 34 shows normal (control) development of the primary bronchus (PB), the secondary bronchi (SB), and the epithelium lining the airways at day four of embryonic development (arrows). MC, mesenchymal cells. Figures 35 and 36: When exposed to high concentration of exogenous FGF-10 (dissolved in matrigel) at day the fourth day of embryonic life, the primary bronchi (PB) and the secondary bronchi (SB) are aberrantly distended (asterisks) and the epithelium lining the airways is unspecified. Figure 37: A toluidine blue-stained section of a matrigel cultured lung at the fourth day of development showing an FGF-10 soaked bead (Bd) which was placed close to the developing lung (the matrigel also had high concentration of dissolved exogenous FGF-10). The secondary bronchi (SB) were bloated (asterisks) but the epithelium which lines the airways (arrows) was well-defined, particularly that associated with the primary bronchus (PB). MC, mesenchymal cells; asterisks, abnormally swollen distal parts of the secondary bronchi; dashed circle, mesenchymal cell proliferation in the area between the FGF-10 bead and the airway epithelium. Scale bars: 100 μm. From unpublished work by JN Maina and B Kramer.

**Figure 11:**
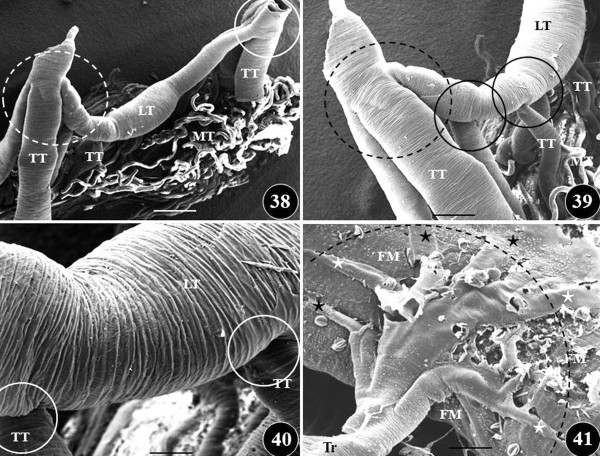
**Figures 38–41: Scanning electron microscope views of the branching pattern of the insectan trachea system, from the grasshopper,*Chrotogonus senegalensis*.** Figures 38–40: In some areas, especially close to the spiracles, many branches originate from a parent segment (dashed circles) (Figures 38, 39) while in other cases, single branches (continuous circles) derive from an original part. TT, transverse trachea; LT, longitudinal trachea; MT, Malphigian tubules. Figure 41: Terminal trachea (Tr) giving rise to many terminal tracheoles (stars) which enter the flight muscle (FM). Dashed arch, shows the expansive part of the flight muscle which is supplied with air by a single terminal trachea. Scale bars: Figure 38, 1.5 mm; Figure 39, 1 mm; Figure 40, 0.5 mm; Figure 41, 0.5 mm. From Maina [318].

**Figure 12:**
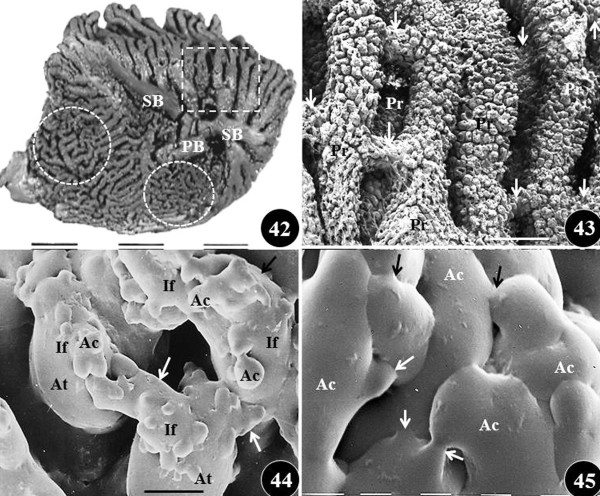
**Figures 42–45: Scanning electron microscope views of the branching pattern and anastomoses of the airways in the mature lung of the domestic fowl,*Gallus gallus*variant*domesticus*.** Figure 42: Medial view of latex cast of the lung showing the different sizes, orientations and anastomoses of the tertiary bronchi (parabronchi). Dashed square, paleopulmonic parabronchi which are long and lie parallel to each other; dashed circles, neopulmonic parabronchi which are short and anastomose profusely; PB, primary bronchus; SB secondary bronchi. Figure 43: Close-up of a stack of cast of paleopulmonic parabronchi (Pr) showing areas which they anastomose (arrows). Figure 44: Close-up of cast of a parabronchus showing atria (At) which give rise to infundibula (If) which anastomose (arrows). AC, air capillaries; dashed lines, interatrial septa. Figure 45: Close-up of cast of air capillaries (AC), the terminal gas exchange units, which profusely anastomose (arrows). Scale bars: Figure 42, 1 cm; Figure 43, 1 mm; Figure 44, 0.5 mm; Figure 45, 8 μm.

Vascular development entails highly complex, well-coordinated processes which include physicochemical stimulators and inhibitors and various gene regulators and signaling molecules (e.g. [307-309]). It is necessary that during lung development, proper juxtaposition occurs between the alveolar surface and the pulmonary capillary endothelial system, forming the blood-gas barrier. It is axiomatic that the development of the vascular system influences the BM of the airways and alveolization [157,310]. Among other organs, the lung has the greatest expression of VEGF (e.g. [311]). Great progress has been made in identifying the signaling pathways which control endothelial cell differentiation and their assembly into a network of cylindrical (tubular) structures with a lumen (e.g. [48,307,308,312]). Lung mesenchyme isn’t uniform in nature. By using histological benchmarks and molecular markers, it has been divided into a sub-mesothelial zone (SMZ) and a sub-epithelial zone (SEZ) [18,241]: Wnt-2a is expressed in the SMZ while Noggin (Nog) is expressed in the SEZ (e.g. [33,142]). Pulmonary vasculature apparently forms between the two mesenchymal compartments [18,241,242]. *In vitro*, 3-D gel preparations have shown that as many as 1,000 different genes are expressed or upregulated during endothelial tubulogenesis (e.g. [48,56,307]). With some of them converting to red blood cells and accumulating haemoglobin (Figure 9), particular mesenchymal cells change to blood cell/blood vessel forming cells (angioblasts), sense the environment, and move by means of long filopodia to surround red blood cells (Figure 10). Progressively, the cells aggregate and demarcating a lumen [308] (Figures 11,12). Members of the VEGF (e.g. [313,314]) and the angiopoetin and the emprin family (e.g. [315-317]) have been associated with the formation of pulmonary vasculature. Transformation, proliferation, and migration of angioblasts is regulated by the local VEGF-A levels and activation of VEGFR-2 [308].

VEGF is a dimeric, heparin-binding glycoprotein. It is an endothelial cell-specific mitogen which initiates cell proliferation and chemotaxis (e.g. [319-322]). By differential mRNA splicing, the VEGF gene generates at least five protein isoforms (VEGF-_122_, -_145_, -_164_, -_188,_ and −_206_) which have different affinities for heparan sulfate as well as for the receptors [VEGFR-1, FLT-1 (fetal liver tyrosinase-1), VEGF-2, FLK-1 (fetal liver kinase-1/KDR)], and neuropilin-1 [323-325]. In different organs, angiogenetic response to VEGF varies. This is dependent on the genetic composition of the animal [326]. VEGF-_122_ doesn’t bind to heparan sulfate and is freely diffusible; VEGF-_188_ is heparin-binding and is mostly associated with the cell surface and the ECM, while; VEGF-_164_ has transitional properties (e.g. [327,328]). Presence of various VEGF ligands and receptors shows specific and redundant regulatory pathways of vascular development (e.g. [329,330]). Mice with an inactivated FLK-1 [313] and -II receptors [326] or VEGF gene die in utero from lack of endothelial cells while knockout ones lack yolk-sac blood-islands and organized blood vessels [321,326]. Inactivation of gene encoding for VEGFR-1 leads to increased number of endothelial cells which block the vessel lumen while that of VEGFR-3 produces abnormally organized blood vessels and causes cardiac failure [326]. Precise control through VEGFR-3 signaling is needed to correct vasculoangiogenesis and hematopoiesis [331]. Gene inactivation experiments show that VEGFR-1 utilizes a negative regulatory effect on VEGFR-2, at least during embryogenesis [326]. Lethality with deletion of a single allele shows the importance of VEGF in embryonic vascular growth [332].

During the development of the lung, airway epithelial cells express VEGF and direct it into the subendothelial matrix while the pulmonary endothelial cells synthesize correct receptors (e.g. [333]). VEGF promotes proliferation, cell mediator migration, angioblast differentiation (in the direction of endothelial cell- lineage), and increases vascular permeability [313,334-336]. The functions are mediated by binding of high-affinity cell receptors and matrix binding sites (e.g. [337,338]). VEGF is vital in *de novo* development of new blood vessels (vasculogenesis) or growth from pre-existing vessels (angiogenesis) (e.g. [189,309,322,323,331,339]). Angiogenesis involves pruning, vessel enlargement, intussusception (vessel splitting), branch remodeling, and extension to form trunks and complex network (e.g. [340,341]). Expression of VEGF gene at the mRNA level is highest in the airway epithelial cells of the lung [342,343], especially in the alveolar type-II epithelial cells [344,345]. Vasculo-epithelial interactions are critical to proper patterning of the airway- and vascular systems (e.g. [346,347]). During the development of the lung, VEGF-A is expressed by the epithelial cells while its primary receptor, VEGFR-2 or FLK-1, is localized in endothelial cells (e.g. [348]). VEGFR-1 and VEGFR-2 expression increases during lung development and accumulates in the pulmonary endothelial cells that lie close to the developing epithelium [116]. HGF (Hepatocyte Growth Factor), a putative endothelial derived factor, mediates reciprocal signaling from the vasculature to the respiratory epithelium [349].

Inhibition of VEGF signaling influences postnatal alveolization [116]. Disruption of the VEGF gene produces mutant embryos with abnormal pulmonary blood vessel development [320,332]. Knockouts for VEGF-A and its two recognized high affinity tyrosine kinase receptors [VEGFR-1 (FLT-1) and VEGFR-2 (KDR/FLK-1)], which are expressed in the primitive vascular endothelium [335,350], die before the lung’s blood capillary plexus forms. Mice overexpressing VEGF in distal epithelial cells present abnormal BM, paucity of acinar buds, impairment of type-I and -II cells, loose mesenchymal mass, and premature development of blood vessels [57,157]. Overexpression of VEGF in the respiratory epithelium leads to excessive vasculogenesis [157,351]. VEGF-_188_ (which is formed in the pulmonary epithelium, especially by the type-II cells) [352] may mediate the convergence and stabilization of the highly organized blood vessel networks that come to be located in the interalveolar wall. VEGF plays different important roles in the repair and maintenance of blood vessels in different pathologies of the mature lung (e.g. [311]). VEGF-A signaling performs an essential part in facilitating communication between the epithelial, mesenchymal, and endothelial parts of the early mouse embryonic lung [310]. It regulates the expression of BMP-4, mSpry-2, mSpry-4, and Sp-c as well as proliferation of both epithelial and mesenchymal compartments. Lazarus et al. [353] showed that blood vessels are requisite for stereotypic 3D epithelial branching and patterning in the lung. They conjectured that inhibition of normal branching, which ensued from vascular loss caused experimentally by ablative methods, could be partly explained by interruption of spatial expression pattern of the branching mediator FGF-10 and by misregulated expression of the branching regulators Shh and Sprouty-2. Del Moral et al. [310] observed that VEGF pathway is involved in driving epithelial to endothelial communication in embryonic mouse lung morphogenesis: VEGF_-164_ stimulates mouse embryonic BM in culture and increases the intensity of the index of proliferation in both epithelium and mesenchyme.

### Transcription Factors (TFs) and other Growth Factors (GFs)

The platelet-derived growth factor (PDGF) is a potent stimulator of cell motility and growth, especially that of connective tissue cells such as fibroblasts and smooth muscle cells (e.g. [138,354,355]). PDGF and its receptor (PDGFR) are expressed in the lung from the onset of the pseudoglandular stage of development [219,356]. Lack of PDGF introduces pulmonary phenotypes that lack alveolar smooth muscle cells and diminished deposition of elastin fibers [137]: PDGF-A and PDGF-Rα are requisite for alveolization [357]. PDGF-Rα positive cells are largely found in the mesenchyme next to the bronchial end-buds [358]. While no distinct lung branching defects were described in PDGF-Rα null mice by Bostrom et al. [137], secondary subdivision didn’t occur in PDGF-A null ones: they exhibited an emphysematous phenotype [357].

FOX [Foxhead Box (Fox)] TFs, also called hepatocyte nuclear factor-3β (HNF-3β), are expressed in the lung [359-362]: they are known to play an important role during lung morphogenesis. FOXA-1 and −2 are co-expressed in the developing lung epithelium while FOXA-1 is correspondingly expressed in the mesenchyme [363]. Silencing FOXA-1 and −2 disrupts BM in the mouse lung, producing a hypoplastic lung with severe defects in epithelial- and smooth muscle cell differentiation [160]. Overexpression of FOXA-2 impairs airway branching, epithelial cell differentiation, and decreases production of surfactant proteins, SP-A, SP-B, and SP-C [364]. Lungs of transgenic mice overexpressing FOXA-2 also display reduced vasculogenesis, possibly from decreased VEGF production by epithelial cells [364].

The GATA family consists of a group of zinc finger domain transcription factors which recognize DNA motif (AT)GATA(A/G) to regulate target gene expression (e.g. [365-367]): they play important roles in regulating cell differentiation during vertebrate development. In the developing lung, GATA-5 and −6 transcription factors are expressed independently [363]: GATA-6 expression is restricted to the respiratory epithelial cells of the developing lung [166] while that of GATA-5 occurs in the smooth muscle cells of the large airways [366]. Furthermore, corresponding to that of SP-A mRNA [165,166,368,369], among the GATA family of zinc finger domain TFs, GATA-6 is expressed before GATA-5. GATA-6 has been shown to regulate specification, differentiation, and maturation of the pulmonary epithelium, branching morphogenesis, and late epithelial cell differentiation [165,252,368,370,371]. Type-II epithelial cells isolated from adult mice and immortalized MLE-15 cells express TTF-1, GATA-6, and various surfactant protein mRNAs [360]. *In vivo*, GATA-6 and Nkx2.1 act in synergistic manner, directing pulmonary epithelial differentiation and development [35]. Inhibition of GATA-6 at E6.0 impeded alveolar maturation and also reduced expression of surfactant proteins which are vital to normal pulmonary function. GATA-6 may play a role in lung development essentially because it regulates expression of TTF-1, which is crucial to lung formation [360,372]. During postnatal alveolization, GATA-6 is not expressed in the developing lung [367]. GATA-6 null mice succumb shortly after implantation, i.e., ~5.5 days after conception [373] and chimeric GATA-6 null ones display a pulmonary phenotype with reduced airway branching [370]. GATA-6 overexpression impairs alveolization [367].

Numbered 1–4, the Notch family consists of four proteins which interact with five ligands (Jagged-1 and −2, and Delta-1, 3, -4) which are expressed on the surface of a neighbouring cell [374]. The greatly conserved Notch/Notch-ligand signaling pathway significantly regulates the development of the lung [48,375].

TTF-1, a member of the Nkx-2 family, is involved in lung development [372,376,377]. TTF-1 promoter activity is directed by combinatorial or cooperative actions of HNF-3 [hepatocyte nuclear factor-3; also known as FOXA (forkhead box A)], Sp (specificity protein)-1, Sp-3, GATA-6, and HOXB-3 (homeobox B-3) TFs [372]. At first expressed in the epithelial cells of dividing lungs, with advancing gestation, TTF-1 expression is considerably reduced and restricted to the type-II cells [169]. In the lung, TTF-1 controls the expression of surfactant proteins that are required for lung stability and lung host defence [372]. Lungs of transgenic mice with increased TTF-1 expression display modest alveolization and type-II cell hyperplasia [378]. Indicating a high degree of conservation, the amino acid sequences of TTF-1 from human, rat, mouse, and other species are very similar [372]. TTF-1 null mice exhibit severe deficiencies of the lung’s BM [155,170]: the bronchial tree is undeveloped while the distal parenchyma is lacking. TTF-1 expression can be activated by other TFs such as FOXA-2 (e.g. [378]).

A number of distinct HOX (homeodomain) TFs are expressed in the developing lung as the mouse embryo gets the end of gestation (e.g. [163,164,379,380]). The HOXB-3 and −4 genes are expressed in the mesenchyme of the trachea, bronchi, and distal lung while HOXA-2 and HOXB-5 are confined to the distal lung mesenchyme, specifying their possible role(s) in BM. The HOXA-5 null mice present defective tracheal structure and defective BM, diminished surfactant production, and thickened alveolar walls [163]. GATA-5 and −6 TFs exhibit non-overlapping spatial expression in the developing lung [365]: GATA-6 expression is restricted to the bronchiolar epithelial cells while GATA-5 is expressed in the smooth muscle cells of the large airways [366].

In humans, Homeobox protein Six1 is a protein which is encoded by the Six-1gene (e.g. [381]). It is a member of the six homeodomain family of TFs [382]. Six-1^(−1-)^ lungs are particularly hypoplastic with considerably reduced epithelial branching and augmented mesenchymal cell density [383]: expressed at the distal epithelial tips of the branching airways and also in the proximate distal mesenchyme, Six-1 coordinates Shh-FGF-10 signaling in embryonic lung to ensure correct levels of proliferation and differentiation of epithelial, mesenchymal, and endothelial cells.

## Summary of molecular regulatory processes in lung development

**FGF-10:** Signals through FGFR-2b. Through instructing Sp-C expression and downregulating expression of BMP-4, FGF-10 regulates differentiation of epithelial cells. Regulatory molecules like other FGFs, Shhs, *β-*catenin (Catnb), and TGF-βs cross-talk with FGF-10-FGFR-2b to tweak lung development: positive regulators of FGF-10 include FOXf-1, Tbx-4, and Tbx-5. Shh inhibits FGF-10 expression but via Gli-3 also controls FOXf-1 availability (Figure 7). By upregulating BMP-4, FGF-10 may influence parabronchial smooth muscle cell development. **FGF-7:** Expressed in the mesenchyme during late stages of lung development and its receptor (FGFR-2) only in the epithelium. Lungs of FGF-7^−/−^ mutant mice are normal. This suggests its redundancy in lung development. FGF-7 activation of FGFR-2b has been shown to regulate interferon-mediated gene expression in adult airway epithelial cell cultures. **FGF-9:** Signals through FGFR-1 and −2. Signaling from the epithelium to the subepithelial mesenchyme, FGF-9 sustains Shh signaling. In a feed-forward loop which maintains mesenchymal FGF sensitivity and mesenchymal Wnt/*β-*catenin signaling, mesenchymal FGF-9 signaling interacts with *β-*catenin mediated Wnt-signaling (Figure 8). FGF-9 (and probably Wnt-7) are two known ligands that can specifically signal from mesothelial (FGF-9) and epithelial cells (FGF-9 and Wnt-7b) to lung mesenchymal FGFRs to control lung development. Regulating mesenchymal proliferation and Wnt-2a expression, mesothelial FGF-9 signals mesenchymal FGFR-1c and FGFR-2c while epithelial FGF-9 predominantly instructs epithelial branching. Overexpression of FGF-9 promptly stops branching morphogenesis (BM). In the submesothelial region, i.e., distal to the source of Shh, FGF-9 induces FGF-10 expression which may promote lengthening of the airways. **FGFR-2c:** Wnt/ *β-*catenin signaling is requisite to activate and sustain expression of FGFR-2c. Sprouty family of genes is one of the key inducible negative regulators of FGFR-2c: FGFR-2b signaling induces expression of Spry-2, a RTK modulator which negatively controls FGF signaling, i.e., it inhibits morphogenesis. The positive feedback loop between FGFR-1, FGFR-2, Wnt-2a, and *β-*catenin (in the mesenchymal cell compartment) countenances input from FGF- and Wnt signaling systems to modulate the output of the whole system, thus coordinating mesenchymal- and epithelial growths. **TGFβ:** Controls lung development through two receptors, TGFRβ-1 and -II, which work in series. TGFβ ligands bind to their associated receptors on the cell surface and activate downstream Smad proteins which translocate into the nucleus and modulate target gene expression. β-integrin and thrombospondin are involved in regulating release of TGFβ mature peptide. **BMP:** Bind to heteromeric complexes of BMP serine/threonine kinase types-1 and -II receptors to activate intracellular signaling pathway. BMP-4 signals to BMPR-1A (ALK 3). Mesenchymal Pod-1 (Tcf-21) and epithelial Wnt signaling regulate BMP-4 which is a well-known target for FGF-10. BMP-4 is believed to control (i.e., to be an antagonist) of FGF-mediated lung bud growth. It probably inhibits distal lung budding through autocrine signaling from the epithelium and can also promote budding in a paracrine manner through unclear mesenchymal signaling. Expression of BMP-4 is controlled by TTF-1. **Shh:** Binds to patched (Ptc), a transmembrane protein, and releases its inhibitory effect on downstream smoothened (Smo), a G-protein coupled transmembrane bridging receptor, leading to activation of cubitus interruptus (Ci). Shh induces Gli gene (Gli-1 and −3) expression which encode transcription factors (TFs) which work downstream of Shh, suppressing FGF-10 expression (Figure 7). Mesenchymal Ptc, Gli-2, and Gli-3 are downregulated in Shh knockdown lung. By directing Hip expression, Shh inhibits FGF-10 expression. **Gli:** Gli-1, -2, and −3, the three vertebrate Ci gene orthologues, are zinc-finger transcription effectors of the Shh signaling pathway. **GATA:** GATA-6 binds to and activates transcription of TTF-1 (Nkx2.1) gene. It also activates expression of different genes involved in respiratory epithelial cell differentiation, including SP-A and SP-C. **Hip-1:** Binds to hedgehog (HH) proteins, moderating HH signaling. Conforming to the expression domains of Ptc-1, Hip-1 is transcriptionally activated in response to HH signaling. Hip-1 and Ptch-1 have redundant roles of instructing airway branching. **Wnt:** Wnt signals are transduced through seven transmembrane-type Wnt receptors encoded by frizzled (Fzd) genes to activate the canonical *β-*catenin-TCF-, the JNK- or the intracellular Ca^2+^-releasing noncanonical pathways. Wnt-2a and Wnt-7b are the canonical Wnt-ligands that activate mesenchymal Wnt/*β-*catenin signaling. Mesenchymal FGF signaling is required for expression of Wnt-2a and for mesenchymal Wnt/*β-*catenin signaling which is vital to sustaining the expression of FGFR-1 and FGFR-2. Wnt-7b- and FGF-9 null mutants exhibit diminished FGF-10 expression. Wnt-5a may antagonize Wnt-7b function of inhibiting lung growth (Figure 7). Receptor internalization-dependent and -independent mechanisms are regulated by Wnt-5a though distinctive pathways. TTF-1, GATA-6, and FOXA-2 TFs, which are critical to lung morphogenesis, regulate Wnt-7b expression (Figure 7). **TGF:** A tyrosine kinase receptor which transfers epidermal growth factor (EGF), TGF signals into the cell. Through EGFR, EGFs positively modulate early lung BM and cytodifferentiation. **VEGF:** VEGF-A, -B, -C, -D, and placental growth factor (PGF) are the VEGF family members. They signal through the cognate receptors - Fetal Liver Kinase-1 [Flk-1/KDR (VEGFR-2), Fetal Liver Tyrosinase-1 [Flt-1 (VEGFR-1)], and Flt-4 (VEGFR-3). Flk-1 positively regulates the VEGF-A signals while Flt-1 negatively regulates the signals. Binding of VEGF-C to VEGFR-3 controls VEGFR-2 signaling. Spatiotemporal expression of Flk-1 and Flt-1 regulates the vascular endothelial cell proliferation and differentiation, inducing vasculogenesis and angiogenesis. VEGF signaling through VEGFR-C occurs synergistically with VEGF-A. VEGF-A induces upregulation of BMP-4 and Sp-C expression. Transcription of VEGF is regulated by hypoxia-inducible TF-1 (HIF-1α) and -2α. **RA:** Its signals are mediated by its nuclear receptors of the steroid hormone receptor superfamily namely retinoic acid receptors (RARs) which include -α, -β, -γ, and δ (i.e., RARα, -β, γ, and δ) and retinoic X receptors α, -β, and -γ (i.e., RXRα, -β, and -γ) which translocate to the nucleus, where they effect gene transcription in target cells. Airway bifurcation is only influenced by the RARα and the RARγ receptors. Together with Tbx genes (in chicks), RA impedes expression and alters distribution of FGF-10 and BMP-4, which must be downregulated in order for BM to happen. Exogenous administration of RA upregulates FOXA-2 and TGFβ-3, two inhibitors of BM. Details are given in the text and can be found mostly in the following detailed reviews: Metzger and Krasnow [25], Perl and Whitsett [11], Roth-Kleiner and Post [116], Cardoso and Lü [40], Lu and Werb [53], De Langhe and Reynolds [117], Affolter et al. [77], Warburton et al. [21], [15,16,18,29,98].

## Molecular aspects of the development of the Avian Lung (Al)

Birds evolved from reptilian stock following mammals (e.g. [8]). Their respiratory system, the parabronchial lung and the air sac system, is remarkably different from the brochioalveolar one of mammals (e.g. [107,132,134,384,385]). Among the air-breathing vertebrates, structurally, the avian lung is purportedly the most complex (e.g. [134,386]) and functionally efficient (e.g. [387-390]) gas exchanger. While the structure of the avian lung has been studied for a long time, e.g., since Coitier [391], compared to the mammalian lung, the genetic and the molecular aspects of its development have been less well-studied. Some of the studies are those by Goldin and Opperman [392] who examined stimulation of DNA synthesis in embryonic chick lung and that of the trachea by the epidermal growth factor (EGF); Chen et al. [393] examined expression and distribution of cell-to-cell adhesion molecules (fibronectin and laminin) on the embryonic chick lung cells; using lectin probes and cationic dyes, Gallanger [394] studied the process of BM; Muraoka et al. [395] examined expression of nuclear factor-kappa-β on epithelial growth and branching of the airways in embryonic chick lung; using tissue recombination experiments, Sakiyama et al. [396] studied the effect of the Tbx-4-FGF-10 system on the separation of the lung bud from the oesophagus and showed that the formation of the airways and the air sacs was caused by region-specific mesenchymal properties and HOXb genes which were expressed in the proximity of the ventral-distal tips of the lung; Stabellini et al. [397] evaluated the roles of polyamines and TGF-β1 on the branching of the airways; Sakiyama et al. [398] found region-specific expression of HOXB-5 to 9 genes, BMP-2, BMP-4, Wnt-5a, and Wnt-11 in the developing respiratory tract of the avian lung; Maina [133,399,400,441] microscopically studied the development of the chicken lung (Figures 13–17) and Maina et al. [231] (Figures 18–21) showed that FGF-2 is expressed and remains upregulated in the epithelial- and mesenchymal cells from very early- to late stages of lung development. In an unpublished study (RG Macharia and JN Maina), it has been observed that Wnt proteins are expressed in the embryo (Figure 22) and at different times and parts of the developing chicken lung (Figures 23–30): together with other morphogenetic factors, the Wnts appear to contribute to the development of the intricate airway- and vascular systems of the avian lung; Miura et al. [401] observed that the development of the air sacs (‘cysts’ as they called them) occurred because of differences in the diffusion of FGF-10 between the dorsal- and the ventral parts of the lung: they attributed the higher dispersal coefficient of the morphogen in the ventral region to relatively loose tissue/cell arrangement in the mesenchyme and the lower one in the dorsal region to greater expression of heparan sulphate proteoglycan (HSPG) which locks in FGF-10: this observation supports the assertion made by, e.g., Kutejova et al. [92] that during lung development, signaling gradient regulates differential gene expression in a concentration-dependent manner; Moura et al. [29] showed that in the embryonic chick lung, expression of FGF-10, FGFR-1 to −4, and Spry-1 was similar to that in the mammalian lung and FGFR inhibition (with FGF receptor agonist SU5402) caused impairment of secondary bronchi (SB) and abnormal lung growth with swollen SB: by *in vitro* tissue culture study (JN Maina and B Kramer, unpublished observations), it was noted that while FGF-10 influences lung development (Figures 31–33), exposure to excessive exogenous level of FGF-10 (dissolved in matrigel and/or soaked in beads) gives similar phenotype, i.e., abnormal (cystic) SB, at expense of the mesenchymal space (Figures 34–37).

## Molecular aspects of development of the insectan tracheal system

Best formed in insects, tracheal respiration has evolved in various animal taxa which include the Onychophora (Peripatus), Solifugae, Phalangidae, some Acarina, Myriapoda, and Chilopoda. The bodies of the tracheates are suffused by air-filled tubes, the trachea. The insectan tracheal system is structurally and functionally remarkable both for its structural design (e.g. [318,402]) and functional efficiency (e.g. [403]). The circulatory- and the respiratory (tracheal) systems are totally disengaged [404,405]: the former plays no meaningful role in gas exchange. Entering through the spiracular openings, oxygen diffuses from the atmosphere to reach the target tissues and cells (e.g. [318,402,406]). The tracheal system in *Drosophila* larvae has afforded a suitable and convenient model for studying the molecular aspects of the development of branched structures [24]. The ‘external factors’ which drive the development of the tracheal system includes the metabolic levels and degrees of hypoxia in the different parts of the body (e.g. [403,407]). Genetic screening of *Drosophila* larvae (e.g. [408-410]) showed that in excess of 200 patterning and morphogenesis genes are involved in the formation of the tracheal system [23]. Some of the genes are involved in the early stages of the development of the tracheal network (primary formation) while others come into effect late to initiate secondary tracheal development. By means of distinct ectodermal placodes (consisting of ~80 cells each) which form on the lateral aspects of the left- and right sides of the embryo, in *D. melanogaster*, trachea start to form at mid-embryogenesis [25,89,408,409,411]. The placodes express the gene (transcription factor) Trachealess [406,411,412] which as per the name, without it, no trachea form. The gene codes for Helix-Loop-Helix-Period Arnt Single-Minded (bHLH-PAS) TF which sequentially regulates transcription of downstream genes that mediate tracheal development [406,411,413]. Each of the placodes invaginates (in- pockets) into the body and then gradually penetrates organs and tissues [24,412,414,415]. The tracheal system develops by BM (e.g. [23,24,89,409,410,416-428]) where the first two branching levels (primary- and secondary trachea) display a stereotypical morphology (Figures 38–40) while the smaller terminal branches (tracheoles), which are very thin extensions, branch profusely (Figure 41), in many tissues possibly contacting practically every single cell in the body.

The repeating pattern of the primary- and the secondary tracheal branches (e.g. [318,402]) shows that an exacting morphogenetic program is controls their development [21,23,25,418]. By means of transcriptional regulation of ‘Trachealess’, all tracheal cells express a *Drosophila* ortholog of the mammalian fibroblast growth factor receptor (FGFR) ‘Breathless (Btl)’ (e.g. [417,429-432]). External to the trachea, in the target tissues, the ligand for this receptor, Branchless (Bnl) (*Drosophila* ortholog of FGF) acts as a chemoattractant of the migrating cells (e.g. [89,418,431]): budding tracheal branches that express Btl migrate towards masses of cells expressing the ligand Bnl. When tracheal cells have reached Bnl-positive cluster of cells, Bnl expression turns-off in that cluster and is instantaneously turned-on a short distance in the path of the advancing branch [88,89]. Quantitatively spatially regulated Btl activity directs tracheal cell migration [431]. High concentration of Bnl provokes expression of pointed (pnt) (a transcription factor) and Sprouty (antagonist of Btl) at the tip of the branch [433]: pointed causes the tip of the tracheal branch to split (forming secondary branches) and Sprouty restricts branching to the tip by inhibiting branching further along the tracheal branch. Expression of Bnl is regulated by the prevailing tissue O2-levels during early larval stages of development [434]. When FGF signaling is extremely upregulated in the entire tracheal tree (by overexpression of a constitutively active form of Btl), many ectopic terminal tracheal branches develop [418]. In *Drosophila*, DWnt-2 gene is involved in tracheal development while wingless (Wg) influences both the developing epidermis and the trachea [51]. DWnt-2 is expressed near the tracheal cells in a pattern different from the Wg one but is also transduced through the canonical Wnt pathway [51]: when the two genes (DWnt-2 and Wg) are deleted, the phenotype is identical or very similar to one observed when the Wnt-pathway is shut down. *Drosophila* Frizzled-2 (Dfz-2) has been identified as a Wg receptor [435]. In *Drosophila*, tracheas don’t start functioning until after fluid in them is cleared at embryonic stage 17 [436].

## Concluding remarks

Insects evolved ~350 million years ago (mya) (e.g. [437]), mammals ~200 mya [8], and birds ~160 mya (e.g. [438]). While their respiratory systems form essentially by branching morphogenesis, a process mechanistically driven by broadly highly conserved genes and molecular factors, in certain important aspects, structural and functional differences exist in the respective gas exchangers, namely the tracheal system, and the bronchioalveolar-, and the parabronchial lungs. For example, in contrast to the dichotomous pattern of branching of particularly the airways in the mammalian lung (e.g. [93,108,115,439]) (Figures 2,3,4,5), in insects, depending on the part of the tracheal system, many as well as single branches stem from original segments (Figures 38–41) and it is even arguable whether the tracheoles, the terminal air conduits (Figure 41), anastomose (e.g. [318,402]): in the avian lung, three morphologically distinctive tiers of airways, namely the primary-, the secondary- and the tertiary bronchi (parabronchi) exist [134,386] and at the parabronchial-, the atrial-, the infundibular, and the air capillary levels, anastomoses occur liberally (Figures 42–45). A fundamental question is why and how actions of congruently conserved genes and molecular factors consequence in different morphologies. Ostensibly, the variations may emanate from spatiotemporal and qualitative and quantitative differences in the patterns of expression of the morphogenetic drivers which specify how long parts lengthen before branching occurs and how branches form. Furthermore, external signals (e.g. hypoxia) may locally modify the morphogenetic programs and regulate upstream and downstream gene expression pathways. Few evolutionally conserved signal transduction pathways are reiteratively exploited during metazoan development [55]. In order to allow more robust systems for regulating a broad spectrum of signaling responses by a limited number of signalling pathways, signals may be integrated at specific connections (nodes) of ‘crosstalk’ between pathways to permit more integrated operations. In nature, economy of structural design (e.g. [440]), which allows systems to perform more by using less, is ubiquitous innovative strategy. More studies are required to identify signaling connections or dedicated protein molecular complexes which may be involved in the integration of the signaling pathways into complex cell signaling networks. It would explain the operational dynamics of genes and molecular factors that lead to different phenotypes. Molecular recursive signalling processes may be hardwired in developmental programs themselves and may be used to craft structural refinements within limits inherent in the conserved systems.

### Ethical

The author’s own research investigations were approved by the University of Witwatersrand’s Animal Ethics Screening Committee.

## Competing interests

The author declares that concerning this manuscript there are no competing interests.
